# Tetra­ethyl­ammonium tris­(thio­cyanato-κ*N*)[tris­(1*H*-pyrazol-1-yl-κ*N* ^2^)methane]­nickelate(II)

**DOI:** 10.1107/S1600536812024774

**Published:** 2012-06-13

**Authors:** Ganna Lyubartseva, Sean Parkin, Uma Prasad Mallik, Hee Kyung Jeon

**Affiliations:** a Department of Chemistry and Physics, Southern Arkansas University, Magnolia, AR 71753, USA; b Department of Chemistry, University of Kentucky, Lexington, KY 40506, USA

## Abstract

The title salt, (C_8_H_20_N)[Ni(NCS)_3_(C_10_H_10_N_6_)], consists of a tetra­ethyl­ammonium cation and an anion comprising an octa­hedral Ni^II^ atom surrounded by three N atoms from a tripodal tris­(pyrazol-1-yl)methane ligand, and three thio­cyanate ligands, each bound at the N-atom end. The ligand Ni—N distances range from 2.097 (2) to 2.127 (2) Å for the tripodal ligand and from 2.045 (2) to 2.075 (2) Å for the thio­cyanate ligands. The dihedral angles between the three pyrazole rings are 59.03 (12), 53.09 (10) and 67.90 (10)°.

## Related literature

For the ligand synthesis, see: Reger *et al.* (2000 ▶). For structural, spectroscopic and angular overlap studies of tris­(pyrazol-1-yl)methane complexes, see: Astley *et al.* (1993 ▶). For literature on tris­(pyrazol-1-yl)borate, see: Czernuszewicz *et al.* (1987 ▶); Kitajima *et al.* (1992 ▶); Lippard & Armstrong (1985 ▶); Lippard *et al.* (1990 ▶). For a related structure, see: Lyubartseva *et al.* (2011 ▶).
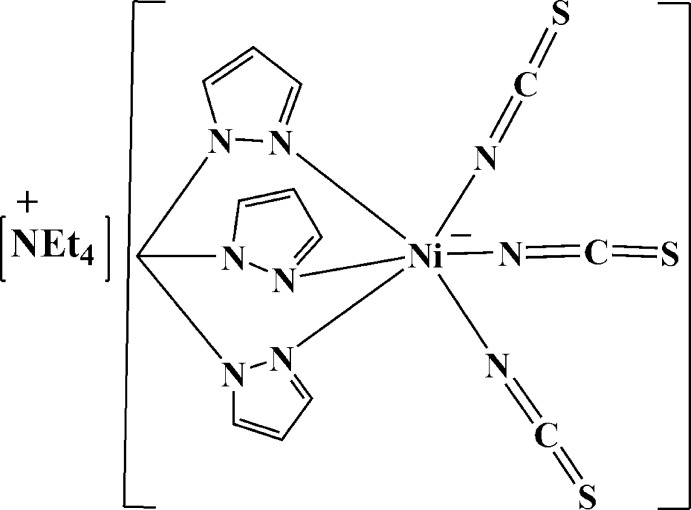


## Experimental

### 

#### Crystal data

(C_8_H_20_N)[Ni(NCS)_3_(C_10_H_10_N_6_)]
*M* *_r_* = 577.44
Monoclinic, 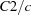

*a* = 31.7117 (12) Å
*b* = 7.4378 (3) Å
*c* = 24.7885 (9) Å
β = 110.592 (2)°
*V* = 5473.2 (4) Å^3^
*Z* = 8
Cu *K*α radiation
μ = 3.41 mm^−1^
*T* = 90 K
0.20 × 0.09 × 0.02 mm

#### Data collection

Bruker X8 Proteum diffractometer
Absorption correction: multi-scan (*SADABS*; Bruker, 2006 ▶) *T*_min_ = 0.562, *T*_max_ = 0.935
17387 measured reflections
17387 independent reflections
16112 reflections with *I* > 2σ(*I*)
*R*_int_ = 0.057

#### Refinement

*R*[*F* ^2^ > 2σ(*F* ^2^)] = 0.054
*wR*(*F* ^2^) = 0.186
*S* = 1.11
17387 reflections
321 parameters
9 restraints
H-atom parameters constrained
Δρ_max_ = 0.65 e Å^−3^
Δρ_min_ = −0.51 e Å^−3^

### 

Data collection: *APEX2* (Bruker, 2006 ▶); cell refinement: *APEX2*; data reduction: *APEX2*; program(s) used to solve structure: *SHELXS97* (Sheldrick, 2008 ▶); program(s) used to refine structure: *SHELXL97* (Sheldrick, 2008 ▶); molecular graphics: *XP* in *SHELXTL* (Sheldrick, 2008 ▶); software used to prepare material for publication: *SHELXL97* and local procedures.

## Supplementary Material

Crystal structure: contains datablock(s) global, I. DOI: 10.1107/S1600536812024774/tk5107sup1.cif

Structure factors: contains datablock(s) I. DOI: 10.1107/S1600536812024774/tk5107Isup2.hkl

Additional supplementary materials:  crystallographic information; 3D view; checkCIF report

## Figures and Tables

**Fig. 1.**
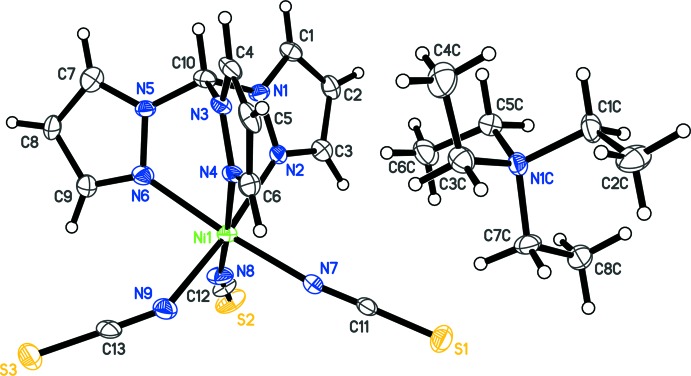
View of the title compound with displacement ellipsoids drawn at the 50% probability level.

**Fig. 2.**
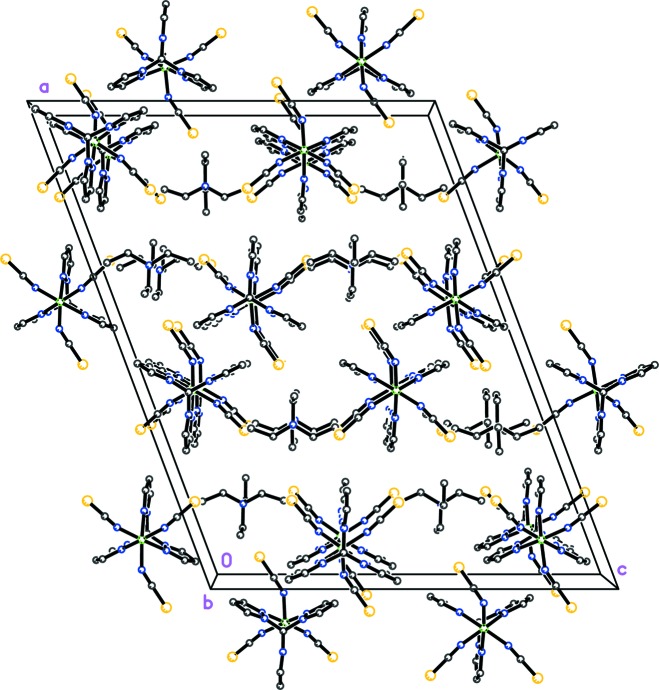
Packing diagram of the title compound as viewed down the b axis. Hydrogen atoms are omitted to enhance clarity.

## supplementary crystallographic information

### Comment

Tris(pyrazol-1-yl)borate has been used to structurally mimic the three histidine residues in the preparation of a hemerythrin analogue (Lippard & Armstrong, 1985, Lippard *et al.*, 1990, Czernuszewicz *et al.*, 1987), as well as to model methane monooxygenase (Kitajima *et al.*, 1992). Tris(pyrazol-1-yl)methane is isoelectronic with tris(pyrazol-1-yl)borate (Astley *et al.*, 1993). While trying to prepare mononuclear [(tpm)Ni^II^*L*_3_]^-1^ , where tpm is tris(pyrazol-1-yl)methane, a symmetrical tridentate neutral nitrogen donor ligand, and *L* is NCS^-^, we obtained [(tpm)_2_Ni^II^][(tpm)Ni^II^(NCS)_3_]_2_.2CH_3_OH as blue monoclinic crystals (Lyubartseva *et al.*, 2011). We hypothesized that by changing the source of nickel and thiocyanate in the reaction, we might be able to synthesize our target compound. Replacement of nickel chloride by commercially available nickel trifluoromethanesulfonate, and tetrabutyl ammonium thiocyanate by tetraethylammonium thiocyanate, the reaction allowed successful isolation of the title complex, tetraethylammonium [tris(1-pyrazolyl)methano tris(thiocyanato) nickelate(II)] as blue monoclinic twinned crystals in moderate yield. Crystallographic analysis of the title complex shows that the structure consists of tetraethylammonium cations and anions consisting of nickel(II) surrounded octahedrally by one tripodal tris(pyrazol-1-yl)methane ligand and three thiocyanate ligands, each bound at the nitrogen end. The tripodal ligand N—Ni distance ranges from 2.097 (2) to 2.127 (2) Å and the distance between N-donor pseudohalide uni-negative anion N—Ni ranges from 2.045 (2) to 2.075 (2) Å, very similar to what we observed before in our previous study (Lyubartseva *et al.*, 2011).

### Experimental

Tris(pyrazolyl)methane ligand was synthesized according to the previously published procedure by Reger *et al.* (2000). Tetraethylammonium thiocyanate and nickel trifluoromethanesulfonate were commercially available and used as received. Ni(OTf)_2_ (179 mg, 0.5 mmol) was dissolved in 35 ml methanol. Tris(pyrazolyl)methane (107 mg, 0.5 mmol) was dissolved in 15 ml methanol. The ligand solution was added drop-wise to the metal containing solution with moderate stirring. Once the addition was complete, tetraethylammonium thiocyanate (0.282 g, 1.5 mmol) was added and stirred for 15 minutes. The clear solution was filtered and methanol was evaporated slowly. Blue crystals were obtained after 1 week (197 mg, 68% yield).

### Refinement

H atoms were found in difference Fourier maps and subsequently placed in idealized positions with constrained distances of 0.98 Å (CH_3_), 1.00 Å (CH), 0.95 Å (C_sp2_H), 0.84 Å (O—H), and with *U*_iso_(H) values set to either 1.2*U*_eq_ or 1.5*U*_eq_ of the attached atom.

The crystal is non-merohedry twinned, in which twin components are related by a 2-fold rotation about the a^*^ axis. The resulting overlap resulted in a large number of rejections during integration, scaling, merging *etc*. Despite many attempts using a range of input parameters, the present dataset was the best that could be obtained.

In response to the low data completeness: The nature of the twinning in this structure (180° rotation about the a^*^ axis) meant that a large number of reflections were rejected at the data reduction stage. After several attempts to eke out more usable reflections by tweaking parameters of the integration (APEX2) and of the scaling and merging (TWINABS), the present dataset was the best that we could manage. Although a complete dataset is of course always preferable, we believe that the structure solution and refinement are unambiguous, and that the model is of a reasonable quality given the unavoidable problems with this structure.

Rigid-body restraints (DELU in *SHELXL97*) were applied to the SCN– groups. The spherical atom scattering factor approximation is known to be particularly bad for carbon atoms involved in triple bonds.

### Crystal data

**Table:**

(C_8_H_20_N)[Ni(NCS)_3_(C_10_H_10_N_6_)]	*F*(000) = 2416
*M**_r_* = 577.44	*D*_x_ = 1.402 Mg m^−^^3^
Monoclinic, *C*2/*c*	Cu *K*α radiation, λ = 1.54178 Å
Hall symbol: -C 2yc	Cell parameters from 9915 reflections
*a* = 31.7117 (12) Å	θ = 3.0–68.1°
*b* = 7.4378 (3) Å	µ = 3.41 mm^−^^1^
*c* = 24.7885 (9) Å	*T* = 90 K
β = 110.592 (2)°	Plate, blue
*V* = 5473.2 (4) Å^3^	0.20 × 0.09 × 0.02 mm
*Z* = 8	

### Data collection

**Table:**

Bruker X8 Proteum diffractometer	17387 independent reflections
Radiation source: fine-focus rotating anode	16112 reflections with *I* > 2σ(*I*)
Graded multilayer optics monochromator	*R*_int_ = 0.057
Detector resolution: 5.6 pixels mm^-1^	θ_max_ = 68.5°, θ_min_ = 3.0°
φ and ω scans	*h* = −38→38
Absorption correction: multi-scan (*SADABS*; Bruker, 2006)	*k* = −8→8
*T*_min_ = 0.562, *T*_max_ = 0.935	*l* = −29→29
17387 measured reflections	

### Refinement

**Table:**

Refinement on *F*^2^	Primary atom site location: structure-invariant direct methods
Least-squares matrix: full	Secondary atom site location: difference Fourier map
*R*[*F*^2^ > 2σ(*F*^2^)] = 0.054	Hydrogen site location: inferred from neighbouring sites
*wR*(*F*^2^) = 0.186	H-atom parameters constrained
*S* = 1.11	*w* = 1/[σ^2^(*F*_o_^2^) + (0.1128*P*)^2^ + 8.5319*P*] where *P* = (*F*_o_^2^ + 2*F*_c_^2^)/3
17387 reflections	(Δ/σ)_max_ = 0.001
321 parameters	Δρ_max_ = 0.65 e Å^−^^3^
9 restraints	Δρ_min_ = −0.51 e Å^−^^3^

### Special details

**Table:**

Experimental. The crystal is twinned by non-merohedry, in which twin components are related by a 2-fold rotation about the a^*^ axis. The resulting overlap resulted in a large number of rejections during integration, scaling, merging *etc*. Despite many attempts using a range of input parameters, the present dataset was the best that we could manage.
Geometry. All e.s.d.'s (except the e.s.d. in the dihedral angle between two l.s. planes) are estimated using the full covariance matrix. The cell e.s.d.'s are taken into account individually in the estimation of e.s.d.'s in distances, angles and torsion angles; correlations between e.s.d.'s in cell parameters are only used when they are defined by crystal symmetry. An approximate (isotropic) treatment of cell e.s.d.'s is used for estimating e.s.d.'s involving l.s. planes.
Refinement. Refinement of *F*^2^ against all reflections. The weighted *R*-value *wR* and goodness of fit *S* are based on *F*^2^. Conventional *R*-values *R* are based on *F*, with *F* set to zero for negative *F*^2^. The threshold expression of *F*^2^ > 2σ(*F*^2^) is used only for calculating *R*-factors(gt) *etc*. and is not relevant to the choice of reflections for refinement. *R*-values based on *F*^2^ are statistically about twice as large as those based on *F*, and *R*-values based on ALL data will be even larger.

### Fractional atomic coordinates and isotropic or equivalent isotropic displacement parameters (Å^2^)

**Table:**

	*x*	*y*	*z*	*U*_iso_*/*U*_eq_	
Ni1	0.407107 (14)	0.17110 (5)	0.63651 (2)	0.01568 (13)	
N1	0.35867 (7)	−0.1867 (3)	0.61187 (10)	0.0153 (5)	
N2	0.35110 (7)	−0.0071 (3)	0.61043 (9)	0.0159 (4)	
C1	0.32006 (8)	−0.2804 (4)	0.58867 (11)	0.0174 (6)	
H1	0.3170	−0.4073	0.5847	0.021*	
C2	0.28630 (9)	−0.1564 (4)	0.57207 (12)	0.0214 (6)	
H2	0.2550	−0.1794	0.5543	0.026*	
C3	0.30707 (9)	0.0117 (4)	0.58641 (11)	0.0191 (6)	
H3	0.2916	0.1236	0.5798	0.023*	
N3	0.42727 (7)	−0.1922 (3)	0.69244 (10)	0.0169 (5)	
N4	0.43242 (7)	−0.0126 (3)	0.70447 (9)	0.0146 (5)	
C4	0.44329 (8)	−0.2906 (4)	0.74113 (12)	0.0176 (6)	
H4	0.4432	−0.4180	0.7436	0.021*	
C5	0.45954 (9)	−0.1741 (4)	0.78610 (13)	0.0219 (7)	
H5	0.4728	−0.2031	0.8258	0.026*	
C6	0.45241 (9)	−0.0008 (4)	0.76109 (11)	0.0195 (6)	
H6	0.4608	0.1085	0.7819	0.023*	
N5	0.42885 (7)	−0.1912 (3)	0.59718 (10)	0.0159 (5)	
N6	0.43478 (8)	−0.0119 (3)	0.59194 (9)	0.0178 (5)	
C7	0.44483 (9)	−0.2883 (4)	0.56257 (12)	0.0212 (6)	
H7	0.4441	−0.4155	0.5587	0.025*	
C8	0.46233 (10)	−0.1675 (4)	0.53401 (14)	0.0246 (7)	
H8	0.4761	−0.1931	0.5065	0.030*	
C9	0.45532 (9)	0.0019 (4)	0.55438 (13)	0.0233 (6)	
H9	0.4643	0.1125	0.5425	0.028*	
C10	0.40484 (8)	−0.2525 (4)	0.63393 (12)	0.0181 (5)	
H10	0.4043	−0.3869	0.6336	0.022*	
N7	0.37742 (8)	0.3308 (3)	0.68012 (11)	0.0202 (5)	
C11	0.35301 (9)	0.4110 (4)	0.69627 (12)	0.0191 (6)	
S1	0.31880 (3)	0.52620 (10)	0.71952 (3)	0.02905 (19)	
N8	0.37895 (8)	0.3221 (3)	0.56293 (11)	0.0228 (6)	
C12	0.35534 (9)	0.3813 (4)	0.52014 (13)	0.0224 (6)	
S2	0.32179 (3)	0.46358 (10)	0.45961 (3)	0.0332 (2)	
N9	0.46528 (8)	0.3251 (3)	0.65824 (11)	0.0214 (5)	
C13	0.49714 (9)	0.3547 (3)	0.64656 (13)	0.0197 (6)	
S3	0.54146 (2)	0.39277 (10)	0.63002 (4)	0.0337 (2)	
N1C	0.33644 (7)	0.0364 (3)	0.85322 (10)	0.0182 (5)	
C1C	0.31210 (10)	−0.0335 (4)	0.89191 (12)	0.0234 (7)	
H1C1	0.3128	−0.1665	0.8916	0.028*	
H1C2	0.2801	0.0038	0.8754	0.028*	
C2C	0.33125 (12)	0.0301 (4)	0.95409 (13)	0.0296 (7)	
H2C1	0.3629	−0.0064	0.9711	0.044*	
H2C2	0.3141	−0.0238	0.9760	0.044*	
H2C3	0.3291	0.1614	0.9553	0.044*	
C3C	0.38609 (9)	−0.0102 (4)	0.87637 (12)	0.0232 (6)	
H3C1	0.4002	0.0498	0.9141	0.028*	
H3C2	0.4003	0.0392	0.8498	0.028*	
C4C	0.39633 (11)	−0.2101 (4)	0.88395 (16)	0.0360 (8)	
H4C1	0.3836	−0.2598	0.9115	0.054*	
H4C2	0.4290	−0.2283	0.8985	0.054*	
H4C3	0.3830	−0.2710	0.8467	0.054*	
C5C	0.31308 (10)	−0.0509 (4)	0.79491 (13)	0.0223 (6)	
H5C1	0.3172	−0.1826	0.7994	0.027*	
H5C2	0.2804	−0.0262	0.7830	0.027*	
C6C	0.32900 (11)	0.0091 (4)	0.74713 (13)	0.0273 (7)	
H6C1	0.3229	0.1377	0.7398	0.041*	
H6C2	0.3130	−0.0590	0.7120	0.041*	
H6C3	0.3615	−0.0125	0.7585	0.041*	
C7C	0.33375 (9)	0.2414 (4)	0.84895 (13)	0.0223 (6)	
H7C1	0.3504	0.2926	0.8875	0.027*	
H7C2	0.3491	0.2817	0.8226	0.027*	
C8C	0.28625 (9)	0.3166 (4)	0.82774 (13)	0.0234 (7)	
H8C1	0.2702	0.2754	0.7883	0.035*	
H8C2	0.2875	0.4483	0.8284	0.035*	
H8C3	0.2703	0.2748	0.8529	0.035*	

### Atomic displacement parameters (Å^2^)

**Table:**

	*U*^11^	*U*^22^	*U*^33^	*U*^12^	*U*^13^	*U*^23^
Ni1	0.0155 (2)	0.0125 (3)	0.0192 (3)	−0.00071 (16)	0.0062 (2)	0.00040 (17)
N1	0.0149 (11)	0.0118 (12)	0.0192 (13)	0.0020 (8)	0.0060 (10)	0.0006 (8)
N2	0.0165 (11)	0.0122 (11)	0.0197 (11)	0.0007 (8)	0.0074 (9)	0.0019 (9)
C1	0.0159 (14)	0.0205 (14)	0.0164 (15)	−0.0059 (10)	0.0062 (11)	−0.0019 (11)
C2	0.0128 (14)	0.0307 (17)	0.0206 (16)	−0.0040 (10)	0.0056 (12)	0.0012 (11)
C3	0.0178 (14)	0.0212 (15)	0.0177 (14)	0.0018 (10)	0.0056 (11)	0.0018 (11)
N3	0.0153 (12)	0.0126 (13)	0.0231 (14)	−0.0025 (8)	0.0071 (10)	0.0016 (9)
N4	0.0112 (12)	0.0126 (13)	0.0183 (12)	−0.0028 (8)	0.0030 (9)	−0.0029 (9)
C4	0.0114 (13)	0.0167 (14)	0.0250 (17)	0.0005 (10)	0.0069 (11)	0.0082 (11)
C5	0.0090 (13)	0.0334 (18)	0.0219 (17)	−0.0029 (10)	0.0038 (11)	0.0032 (11)
C6	0.0128 (13)	0.0234 (16)	0.0226 (16)	−0.0012 (10)	0.0066 (11)	−0.0011 (12)
N5	0.0128 (11)	0.0120 (12)	0.0228 (14)	0.0002 (8)	0.0062 (10)	0.0006 (8)
N6	0.0211 (13)	0.0152 (13)	0.0194 (13)	−0.0011 (9)	0.0101 (10)	0.0013 (9)
C7	0.0206 (14)	0.0248 (16)	0.0189 (16)	0.0040 (11)	0.0078 (12)	−0.0004 (11)
C8	0.0270 (16)	0.0271 (18)	0.0283 (19)	0.0036 (11)	0.0202 (14)	−0.0006 (12)
C9	0.0192 (15)	0.0238 (16)	0.0288 (16)	0.0005 (11)	0.0107 (12)	0.0018 (12)
C10	0.0138 (13)	0.0166 (14)	0.0205 (15)	0.0003 (10)	0.0017 (11)	0.0022 (11)
N7	0.0249 (13)	0.0151 (13)	0.0248 (15)	−0.0019 (9)	0.0139 (11)	0.0000 (9)
C11	0.0230 (15)	0.0143 (14)	0.0197 (15)	−0.0039 (11)	0.0072 (11)	0.0030 (11)
S1	0.0347 (4)	0.0252 (4)	0.0350 (4)	0.0087 (3)	0.0220 (4)	0.0034 (3)
N8	0.0226 (13)	0.0137 (14)	0.0290 (16)	0.0033 (9)	0.0051 (11)	0.0009 (10)
C12	0.0261 (15)	0.0141 (14)	0.0282 (17)	0.0021 (11)	0.0112 (13)	−0.0052 (12)
S2	0.0456 (5)	0.0246 (4)	0.0237 (4)	0.0145 (3)	0.0052 (3)	0.0027 (3)
N9	0.0187 (12)	0.0164 (12)	0.0294 (15)	−0.0015 (8)	0.0088 (11)	0.0029 (9)
C13	0.0185 (14)	0.0109 (13)	0.0265 (17)	0.0031 (10)	0.0039 (12)	0.0048 (11)
S3	0.0239 (4)	0.0253 (5)	0.0565 (6)	0.0003 (3)	0.0200 (4)	0.0070 (4)
N1C	0.0157 (12)	0.0221 (14)	0.0189 (12)	0.0017 (9)	0.0085 (10)	0.0018 (10)
C1C	0.0227 (16)	0.0275 (17)	0.0251 (16)	0.0032 (12)	0.0148 (13)	0.0081 (12)
C2C	0.047 (2)	0.0178 (17)	0.0262 (17)	0.0053 (13)	0.0162 (15)	0.0043 (12)
C3C	0.0166 (14)	0.0280 (16)	0.0255 (16)	0.0018 (11)	0.0082 (12)	0.0029 (12)
C4C	0.0287 (18)	0.0391 (19)	0.042 (2)	0.0134 (14)	0.0152 (15)	0.0140 (15)
C5C	0.0219 (16)	0.0215 (16)	0.0253 (17)	−0.0027 (12)	0.0105 (13)	−0.0006 (11)
C6C	0.0346 (18)	0.0257 (17)	0.0245 (16)	−0.0059 (12)	0.0140 (14)	−0.0014 (13)
C7C	0.0286 (15)	0.0128 (15)	0.0268 (16)	−0.0011 (11)	0.0114 (13)	−0.0013 (11)
C8C	0.0218 (15)	0.0216 (17)	0.0287 (18)	0.0039 (11)	0.0114 (13)	0.0034 (12)

### Geometric parameters (Å, º)

**Table:**

Ni1—N7	2.045 (2)	N7—C11	1.155 (4)
Ni1—N8	2.059 (2)	C11—S1	1.638 (3)
Ni1—N9	2.075 (2)	N8—C12	1.149 (4)
Ni1—N4	2.097 (2)	C12—S2	1.623 (3)
Ni1—N2	2.126 (2)	N9—C13	1.166 (4)
Ni1—N6	2.127 (2)	C13—S3	1.621 (3)
N1—C1	1.349 (3)	N1C—C3C	1.514 (3)
N1—N2	1.356 (3)	N1C—C5C	1.519 (4)
N1—C10	1.456 (3)	N1C—C1C	1.519 (3)
N2—C3	1.319 (3)	N1C—C7C	1.529 (4)
C1—C2	1.363 (4)	C1C—C2C	1.520 (4)
C1—H1	0.9500	C1C—H1C1	0.9900
C2—C3	1.400 (4)	C1C—H1C2	0.9900
C2—H2	0.9500	C2C—H2C1	0.9800
C3—H3	0.9500	C2C—H2C2	0.9800
N3—C4	1.349 (3)	C2C—H2C3	0.9800
N3—N4	1.366 (3)	C3C—C4C	1.519 (4)
N3—C10	1.444 (4)	C3C—H3C1	0.9900
N4—C6	1.324 (3)	C3C—H3C2	0.9900
C4—C5	1.362 (4)	C4C—H4C1	0.9800
C4—H4	0.9500	C4C—H4C2	0.9800
C5—C6	1.414 (4)	C4C—H4C3	0.9800
C5—H5	0.9500	C5C—C6C	1.509 (4)
C6—H6	0.9500	C5C—H5C1	0.9900
N5—C7	1.349 (4)	C5C—H5C2	0.9900
N5—N6	1.359 (3)	C6C—H6C1	0.9800
N5—C10	1.451 (3)	C6C—H6C2	0.9800
N6—C9	1.315 (4)	C6C—H6C3	0.9800
C7—C8	1.376 (4)	C7C—C8C	1.517 (4)
C7—H7	0.9500	C7C—H7C1	0.9900
C8—C9	1.404 (4)	C7C—H7C2	0.9900
C8—H8	0.9500	C8C—H8C1	0.9800
C9—H9	0.9500	C8C—H8C2	0.9800
C10—H10	1.0000	C8C—H8C3	0.9800
			
N7—Ni1—N8	90.83 (9)	N3—C10—H10	108.7
N7—Ni1—N9	94.28 (10)	N5—C10—H10	108.7
N8—Ni1—N9	89.87 (10)	N1—C10—H10	108.7
N7—Ni1—N4	94.08 (9)	C11—N7—Ni1	166.6 (2)
N8—Ni1—N4	172.38 (9)	N7—C11—S1	179.4 (3)
N9—Ni1—N4	95.57 (9)	C12—N8—Ni1	164.9 (2)
N7—Ni1—N2	91.84 (9)	N8—C12—S2	179.6 (3)
N8—Ni1—N2	90.86 (9)	C13—N9—Ni1	144.0 (2)
N9—Ni1—N2	173.83 (9)	N9—C13—S3	179.2 (3)
N4—Ni1—N2	83.16 (8)	C3C—N1C—C5C	110.9 (2)
N7—Ni1—N6	175.50 (9)	C3C—N1C—C1C	112.0 (2)
N8—Ni1—N6	91.07 (9)	C5C—N1C—C1C	105.6 (2)
N9—Ni1—N6	89.81 (9)	C3C—N1C—C7C	106.25 (19)
N4—Ni1—N6	83.63 (8)	C5C—N1C—C7C	111.3 (2)
N2—Ni1—N6	84.05 (9)	C1C—N1C—C7C	110.9 (2)
C1—N1—N2	111.9 (2)	N1C—C1C—C2C	115.0 (3)
C1—N1—C10	128.9 (2)	N1C—C1C—H1C1	108.5
N2—N1—C10	119.13 (19)	C2C—C1C—H1C1	108.5
C3—N2—N1	105.4 (2)	N1C—C1C—H1C2	108.5
C3—N2—Ni1	135.22 (18)	C2C—C1C—H1C2	108.5
N1—N2—Ni1	119.00 (15)	H1C1—C1C—H1C2	107.5
N1—C1—C2	106.1 (2)	C1C—C2C—H2C1	109.5
N1—C1—H1	126.9	C1C—C2C—H2C2	109.5
C2—C1—H1	126.9	H2C1—C2C—H2C2	109.5
C1—C2—C3	106.2 (2)	C1C—C2C—H2C3	109.5
C1—C2—H2	126.9	H2C1—C2C—H2C3	109.5
C3—C2—H2	126.9	H2C2—C2C—H2C3	109.5
N2—C3—C2	110.4 (2)	N1C—C3C—C4C	114.8 (2)
N2—C3—H3	124.8	N1C—C3C—H3C1	108.6
C2—C3—H3	124.8	C4C—C3C—H3C1	108.6
C4—N3—N4	110.9 (2)	N1C—C3C—H3C2	108.6
C4—N3—C10	128.9 (2)	C4C—C3C—H3C2	108.6
N4—N3—C10	120.1 (2)	H3C1—C3C—H3C2	107.5
C6—N4—N3	105.8 (2)	C3C—C4C—H4C1	109.5
C6—N4—Ni1	135.53 (18)	C3C—C4C—H4C2	109.5
N3—N4—Ni1	118.63 (16)	H4C1—C4C—H4C2	109.5
N3—C4—C5	107.6 (2)	C3C—C4C—H4C3	109.5
N3—C4—H4	126.2	H4C1—C4C—H4C3	109.5
C5—C4—H4	126.2	H4C2—C4C—H4C3	109.5
C4—C5—C6	105.4 (3)	C6C—C5C—N1C	115.8 (2)
C4—C5—H5	127.3	C6C—C5C—H5C1	108.3
C6—C5—H5	127.3	N1C—C5C—H5C1	108.3
N4—C6—C5	110.3 (2)	C6C—C5C—H5C2	108.3
N4—C6—H6	124.8	N1C—C5C—H5C2	108.3
C5—C6—H6	124.8	H5C1—C5C—H5C2	107.4
C7—N5—N6	111.7 (2)	C5C—C6C—H6C1	109.5
C7—N5—C10	128.9 (2)	C5C—C6C—H6C2	109.5
N6—N5—C10	119.3 (2)	H6C1—C6C—H6C2	109.5
C9—N6—N5	105.3 (2)	C5C—C6C—H6C3	109.5
C9—N6—Ni1	135.67 (19)	H6C1—C6C—H6C3	109.5
N5—N6—Ni1	118.83 (16)	H6C2—C6C—H6C3	109.5
N5—C7—C8	106.7 (2)	C8C—C7C—N1C	114.6 (2)
N5—C7—H7	126.7	C8C—C7C—H7C1	108.6
C8—C7—H7	126.7	N1C—C7C—H7C1	108.6
C7—C8—C9	104.9 (3)	C8C—C7C—H7C2	108.6
C7—C8—H8	127.5	N1C—C7C—H7C2	108.6
C9—C8—H8	127.5	H7C1—C7C—H7C2	107.6
N6—C9—C8	111.4 (3)	C7C—C8C—H8C1	109.5
N6—C9—H9	124.3	C7C—C8C—H8C2	109.5
C8—C9—H9	124.3	H8C1—C8C—H8C2	109.5
N3—C10—N5	110.5 (2)	C7C—C8C—H8C3	109.5
N3—C10—N1	110.4 (2)	H8C1—C8C—H8C3	109.5
N5—C10—N1	109.8 (2)	H8C2—C8C—H8C3	109.5
			
C1—N1—N2—C3	0.5 (3)	N9—Ni1—N6—N5	138.72 (19)
C10—N1—N2—C3	177.3 (2)	N4—Ni1—N6—N5	43.10 (17)
C1—N1—N2—Ni1	−173.73 (18)	N2—Ni1—N6—N5	−40.65 (18)
C10—N1—N2—Ni1	3.1 (3)	N6—N5—C7—C8	0.6 (3)
N7—Ni1—N2—C3	49.5 (3)	C10—N5—C7—C8	176.2 (2)
N8—Ni1—N2—C3	−41.3 (3)	N5—C7—C8—C9	0.0 (3)
N4—Ni1—N2—C3	143.4 (3)	N5—N6—C9—C8	1.0 (3)
N6—Ni1—N2—C3	−132.3 (3)	Ni1—N6—C9—C8	−173.5 (2)
N7—Ni1—N2—N1	−138.4 (2)	C7—C8—C9—N6	−0.6 (3)
N8—Ni1—N2—N1	130.8 (2)	C4—N3—C10—N5	−122.9 (3)
N4—Ni1—N2—N1	−44.47 (19)	N4—N3—C10—N5	60.8 (3)
N6—Ni1—N2—N1	39.8 (2)	C4—N3—C10—N1	115.4 (3)
N2—N1—C1—C2	−0.5 (3)	N4—N3—C10—N1	−60.9 (3)
C10—N1—C1—C2	−176.9 (3)	C7—N5—C10—N3	125.4 (3)
N1—C1—C2—C3	0.2 (3)	N6—N5—C10—N3	−59.4 (3)
N1—N2—C3—C2	−0.4 (3)	C7—N5—C10—N1	−112.6 (3)
Ni1—N2—C3—C2	172.5 (2)	N6—N5—C10—N1	62.7 (3)
C1—C2—C3—N2	0.1 (3)	C1—N1—C10—N3	−125.4 (3)
C4—N3—N4—C6	0.9 (3)	N2—N1—C10—N3	58.4 (3)
C10—N3—N4—C6	177.8 (2)	C1—N1—C10—N5	112.5 (3)
C4—N3—N4—Ni1	−176.83 (17)	N2—N1—C10—N5	−63.7 (3)
C10—N3—N4—Ni1	0.1 (3)	N8—Ni1—N7—C11	57.8 (9)
N7—Ni1—N4—C6	−42.9 (3)	N9—Ni1—N7—C11	147.7 (9)
N9—Ni1—N4—C6	51.8 (3)	N4—Ni1—N7—C11	−116.4 (9)
N2—Ni1—N4—C6	−134.3 (3)	N2—Ni1—N7—C11	−33.1 (9)
N6—Ni1—N4—C6	141.0 (3)	N7—Ni1—N8—C12	−85.6 (9)
N7—Ni1—N4—N3	133.94 (18)	N2—Ni1—N8—C12	6.2 (9)
N9—Ni1—N4—N3	−131.34 (18)	N6—Ni1—N8—C12	90.3 (9)
N2—Ni1—N4—N3	42.58 (18)	N7—Ni1—N9—C13	−163.3 (3)
N6—Ni1—N4—N3	−42.16 (17)	N8—Ni1—N9—C13	−72.5 (3)
N4—N3—C4—C5	−0.3 (3)	N4—Ni1—N9—C13	102.2 (3)
C10—N3—C4—C5	−176.9 (2)	N6—Ni1—N9—C13	18.6 (3)
N3—C4—C5—C6	−0.3 (3)	C3C—N1C—C1C—C2C	56.6 (3)
N3—N4—C6—C5	−1.1 (3)	C5C—N1C—C1C—C2C	177.4 (3)
Ni1—N4—C6—C5	176.07 (18)	C7C—N1C—C1C—C2C	−61.9 (3)
C4—C5—C6—N4	0.9 (3)	C5C—N1C—C3C—C4C	−58.8 (3)
C7—N5—N6—C9	−1.0 (3)	C1C—N1C—C3C—C4C	58.8 (3)
C10—N5—N6—C9	−177.0 (2)	C7C—N1C—C3C—C4C	−180.0 (3)
C7—N5—N6—Ni1	174.60 (18)	C3C—N1C—C5C—C6C	−63.1 (3)
C10—N5—N6—Ni1	−1.4 (3)	C1C—N1C—C5C—C6C	175.4 (3)
N8—Ni1—N6—C9	42.5 (3)	C7C—N1C—C5C—C6C	55.0 (3)
N9—Ni1—N6—C9	−47.3 (3)	C3C—N1C—C7C—C8C	−178.6 (2)
N4—Ni1—N6—C9	−143.0 (3)	C5C—N1C—C7C—C8C	60.6 (3)
N2—Ni1—N6—C9	133.3 (3)	C1C—N1C—C7C—C8C	−56.7 (3)
N8—Ni1—N6—N5	−131.41 (19)		
